# Structure of a lectin from *Canavalia gladiata *seeds: new structural insights for old molecules

**DOI:** 10.1186/1472-6807-7-52

**Published:** 2007-08-02

**Authors:** Plínio Delatorre, Bruno AM Rocha, Emmanuel P Souza, Taianá M Oliveira, Gustavo A Bezerra, Frederico BMB Moreno, Beatriz T Freitas, Tatiane Santi-Gadelha, Alexandre H Sampaio, Walter F Azevedo, Benildo S Cavada

**Affiliations:** 1 Departamento de Bioquímica e Biologia Molecular, Universidade Federal do Ceará, Ceará, Brazil; 2 Departamento de Biologia, Universidade Regional do Cariri, Ceará, Brazil; 3 Departamento de Biologia, Universidade Federal da Paraíba, Paraíba, Brazil; 4 Departamento de Física, IBILCE, Universidade Estadual Paulista, São Paulo, Brazil; 5 Faculdade de Biociências, PUCRS, Av. Ipiranga 6681, Zip Code 90619-900, Porto Alegre, RS, Brazil

## Abstract

**Background:**

Lectins are mainly described as simple carbohydrate-binding proteins. Previous studies have tried to identify other binding sites, which possible recognize plant hormones, secondary metabolites, and isolated amino acid residues. We report the crystal structure of a lectin isolated from *Canavalia gladiata *seeds (CGL), describing a new binding pocket, which may be related to pathogen resistance activity in ConA-like lectins; a site where a non-protein amino-acid, α-aminobutyric acid (Abu), is bound.

**Results:**

The overall structure of native CGL and complexed with α-methyl-mannoside and Abu have been refined at 2.3 Å and 2.31 Å resolution, respectively. Analysis of the electron density maps of the CGL structure shows clearly the presence of Abu, which was confirmed by mass spectrometry.

**Conclusion:**

The presence of Abu in a plant lectin structure strongly indicates the ability of lectins on carrying secondary metabolites. Comparison of the amino acids composing the site with other legume lectins revealed that this site is conserved, providing an evidence of the biological relevance of this site. This new action of lectins strengthens their role in defense mechanisms in plants.

## Background

Most of the biochemical lectin studies have been based on monochromatic view since almost all the properties of these proteins had been commonly reported in terms of lectin-carbohydrate recognition. For several years, the definition of lectins has been improving focused on the carbohydrate-binding properties. The most recent accepted definition establishes lectins as proteins with at least one non-catalytic domain able to recognize and bind reversibly to specific mono and oligosaccharides. They are subdivided into four types: merolectins, hololectins, chimerolectins and superlectins. This classification was conceived in terms of the carbohydrate-binding domain and another unrelated domain [1]. Several studies have tried to find other binding sites that could possibly recognize plant hormones, secondary metabolites and isolated amino acid residues [2-4].

Over 250 non-protein amino acids have been identified in plants [5]. A number of these compounds are intermediates in the synthesis and catabolism of protein amino acids [6]. However, many of these non-protein amino acids may play a role as defensive agents. They show their toxicity in many ways; some of them block the synthesis and the absorption of protein amino acids or can wrongly incorporated into proteins in organisms that feed on these plants. Plants that synthesize non-protein amino acids are not susceptible to the toxicity of these compounds. Seeds from *Canavalia ensiformis*, which synthesize high quantities of non-protein amino acids, display a biological system capable of discriminating between these amino acids and the others [7].

Non-protein amino acids are especially abundant in Leguminosae, Liliaceae and in several higher fungi and marine algae. Plant organs rich in these metabolites are seeds (Leguminosae) or rhizomes (Liliaceae). Concentrations in seeds can exceed 10% of dry weight and up to 50% of the nitrogen could be attributed to them. Since non-protein amino acids are often remobilized during germination, they certainly function as N-storage compounds in addition to their role as defense chemicals [8]. If non-protein amino acids are taken up by herbivores, microorganisms or other plants, they may interfere with their metabolism.

Aminobutyric acid (Abu) is a non-protein amino acid that can protect certain plants against pathogens; for instance, when introduced into Arabidopis plants, it has the ability to induce resistance to certain pathogens. Abu protects these plants against pathogens through the activation of natural defense mechanisms of the plant, such as callose deposition, hypersensitive response (HR), and the formation of trailing necroses. Induced resistance is often associated with a process called priming, which is an increased capacity to mobilize cellular defense responses [9].

Most plant lectins not only play a role in the plant itself (e.g., as a store of nitrogen or as a specific recognition factor) but are also capable of interfering with the functioning of foreign organisms through an interaction with glycoconjugates on the surface or in the digestive tract of these organisms [10]. Although this interference has been reported as a specific event of carbohydrate recognition, it has not been elucidated yet. Stress-regulated pathways for rapid and high gene expression are one of the essential elements in stress acclimation. Salicylic acid, jasmonic acid, systemin, ethylene, and aminobutyric acid have been implicated in the potentiation of gene expression [11,12], and other signal molecules have been shown to play a similar role [9].

The content of free protein amino acids in seeds varies among species and increases dramatically after germination. More non-protein amino acids were found in lentil seedlings compared to the seeds [12]. Most plant lectins are probably involved in plant defense [13]. The mechanism of action still remains unclear even though induced response mechanisms are proposed for many pathogen-mediated injuries in plants. The direct interference with viruses and microorganisms is rather exceptional, and the deleterious effects of plant lectins on both predatory invertebrates and animals are well documented [13].

We report here the crystal structure of a native and complexed lectin isolated from *Canavalia gladiata *seeds, describing a new binding pocket in ConA-like lectins, which may be related to pathogen resistance, a site where a non-protein amino acid, such as α-amino butyric acid, can bind.

## Results and discussion

### Overall structure of CGL

**Figure 1:**
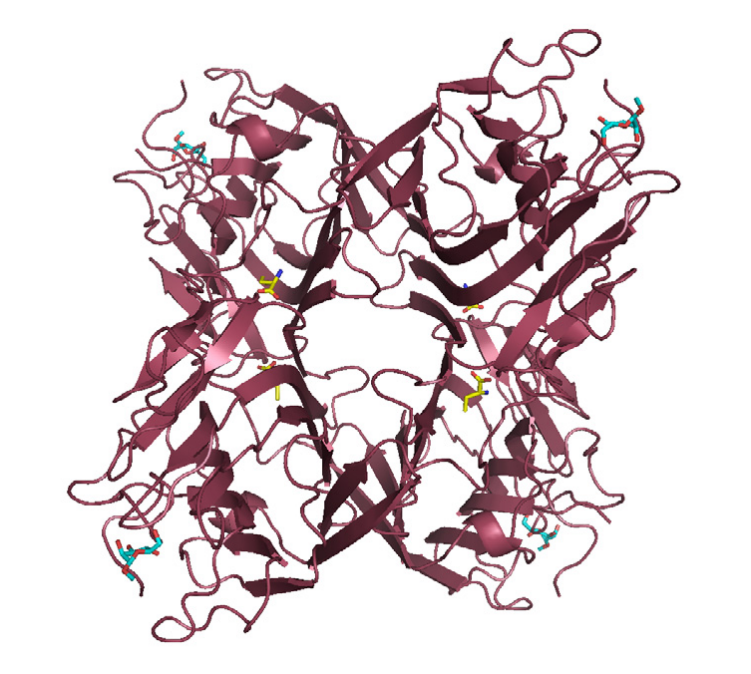
**Crystal structure of CGL-αMM**. The vision of the Abu position can be seen in the canonical dimmer between the monomers at hydrophobic cavity. Abu is displayed in yellow and α-methyl-mannoside is in blue.

The crystal structure of native CGL and in complex with α-methyl-mannoside (CGL-αMM) provides a new source of information for the understanding of lectins and reveals a new binding site for a non protein amino acid at the monomers contact interface of each canonical dimer of leguminous lectins. The overall structure of native CGL and CGL-αMM has been refined at the 2.3 Å and 2.31 Å resolutions, respectively (Figure 1). The lectin CGL model was refined in the absence of ligand to produce the Fo-Fc map. This map is unbiased insofar as the atomic coordinates used for phase calculations have never been refined together with the ligand. The ligand was finally inserted to produce the final coordinates after some further refinement. The models present good stereochemistry, and the Ramachandran plot is consistent with a geometrically well-defined structure (Table 1).

**Table 1:** Statistics of data collection, refinement and quality of the structure

	CGL	CGL-αMM
**Data collection**		
Total number of observations	238,197	483,177
Total number of unique observations	57,503	61,808
*Rmerge *(%)	8.8 (34.4)	7.3 (35.4)
Resolution limit (Å)	42.64-2.3	51.99-2.31
Completeness (%)	97.87 (98.2)	99.1 (94.5)
Multiplicity	3.9	4.5
(I)/σ	7.2 (2.2)	8.9 (2.0)
Wavelength (Å)	1.431	1.431
Space group	*C2221*	*C2221*
Cell parameters (Å)	a = 100.90	a = 100.91
	b = 115.35	b = 115.75
	c = 241.08	c = 241.62
		
**Refinement**		
Resolution range (Å)	10-2.3	9.99-2.31
*Rfactor *(%)	18.38	16.87
*Rfree *(%)	23.28	22.31
Number amino acid residues in biological assembly	948	948
Number of water molecules	432	448
RMS deviations from ideal values		
Bond lengths (Å)	0.021	0.026
Bond angles (degrees)	2.019	2.223
Temperature factors		
Average B value for whole protein chain (Å ^2^)	20.6	20.1
Ramachandran plot		
Residues in most favored regions	84.0	86.2
Residues in additional allowed regions	15.3	13.2
Residues in generously allowed regions	0.7	0.6

*Values in parenthesis represent the high resolution shell.

Among the leguminous lectins isolated from the tribe Diocleinae, only those purified from the seeds of *C. ensiformis*, *C. brasiliensis*, *C. maritima*, *Dioclea grandiflora*, *D. guianensis *and *Cratylia mollis *have three-dimensional structures resolved by X-ray crystallography [14,15]. These lectins showed respectively 98%, 98%, 99%, 84%, 82% and 81% similarity in their amino acid sequences as compared with CGL.

The oligomeric structures of CGL and CGL-αMM involve two 'canonical' dimers, and these dimers are associated by salt bridges between β-strands. The model was examined by Xfit, and one region around Asp 139 showed a well-defined electron density (fo-fc contoured at 5σ), not elucidated for the model, the atomic coordinates for this structure were deposited without the elucidation of this density with PDB access 1 WUV. After an analysis of the amino acid and secondary metabolite content in leguminous seeds, α-aminobutyric acid (Abu), a non-protein amino acid, was chosen according to electron density. The atomic coordinates for the structure in complex with Abu and α-methyl-mannoside (CGL-αMM) was deposited in PDB with access code 2D7F.

An interesting feature of the presence of the Abu molecule in CGL structures is the great increase in electron density in loops. Previous ConA structures showed some poor density regions at N-terminal residues and in surface loops at residues 118–122, 149–151, 160–163 [16-18]. The CGL structure presents well-defined electron density in these loops and is the first ConA-like structure that shows a stable loop in 118–122 region.

In both CGL crystal structures, the amino acids involved in metal binding are conserved. The calcium metal ion bond drives the trans-to-cis isomerization of the Ala207-Asp208 peptide bond, which is conserved in all known legume lectin crystal structures. This cis-peptide bond contributes to the stabilization of the binding pocket by orienting the positions of residues Asn14 and Arg228.

The sugar-binding site is a highly conserved region in ConA-like lectins. The role of conserved amino acid residues in monosaccharide interactions with lectins has been extensively reported. Commonly, five to eight H-bonds occur between monosaccharides and the Diocleinae lectin domain amino acids Asn14, Leu99, Tyr100, Asp208 and Arg228 [19]; CGL-αMM displays an alpha-methyl-mannoside recognition involving seven H-bonds with specific amino acid residues and another with a water molecule. The nitrogen side chain of Asn14 forms a single H-bond with αMM O4 oxygen at 2.6 Å; the main chain nitrogens of Leu99 and Tyr100 are connected with αMM O5 and O6 oxygens. O5 is at 3.0 Å from Leu99 and O6 is at 2.9 Å and 2.98 Å from Leu99 and Tyr100, respectively. Oxygens from Asp 208 side chain are the CGL closest atoms from the αMM. They are positioned at 2.5 Å from O7 and 2.7 Å from O6, and the main chain nitrogen of Arg 228 makes a 2.9 Å H-bond with αMM O3 oxygen.

### α-aminobutyric acid (Abu) interaction

**Figure 2:**
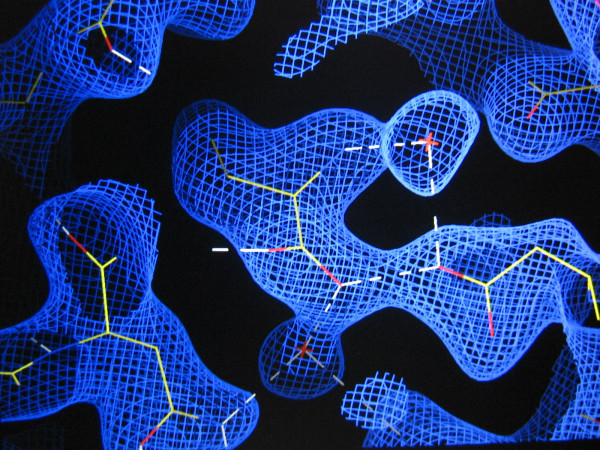
**CGL hydrophobic pocket**. Abu electron density (2Fo-Fc) map countered at 1 σ.

**Figure 3:**
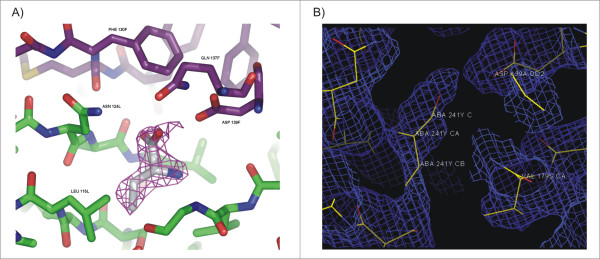
**Abu electron density in hydrophobic pocket**. (A) Fo-Fc omit map of Abu in CGL structure. (B) Fo-Fc electron density map of CGL structure solved at room temperature (without glycerol as cryoprotectant) at 3.0 Å

Abu was found at the monomers contact interface of CGL-αMM (Figure 2). The Fo-Fc omit map in figure 3a is contoured at 3σ cutoff and confirms that the map is unbiased and the electron density clearly has Abu fitted in the map. Abu ligand is accessible to the solvent surface by the carboxyl group and interacts with one chain through hydrogen bond with Asp139 and an interstitial water molecule, which interacts with Asn 124 and with the other chain through hydrogen bonds with Ala 125 and with another interstitial water interacting with Gln 137, and through hydrophobic interaction with Leu126 and Val 179 (Figure 2). The presence of Abu at the monomers interface increased the intermolecular contacts and strongly stabilized the canonical dimers. This interaction decreased the vibrational spectrum and increased the X-ray scattering in loop regions. In order to obtain further evidence for the presence of ABU in the structure, we collected data in the absence of glycerol at room temperature. Analysis of these low resolution CGL structures (solved at approximately 3.0 Å resolution), also indicates presence of the electron density for the Abu. At room temperature, protein crystals were mounted in sealed thin-walled glass capillary tubes for X-ray data collection, these conditions lead to a loss in the resolution power. The best data set diffracted to 3.0 Å and shows ABU in a non-conclusive electron density map, but the electron density calculated at 2.3 Å shows clearly that the map corresponding to the ABU density. Due to the distance of atoms is really difficult to consider that this density refers to a set of ordered waters in both structures. The structure solved in the absence of glycerol and showing appropriate density for ABU is shown in figure 3b.

**Figure 4:**
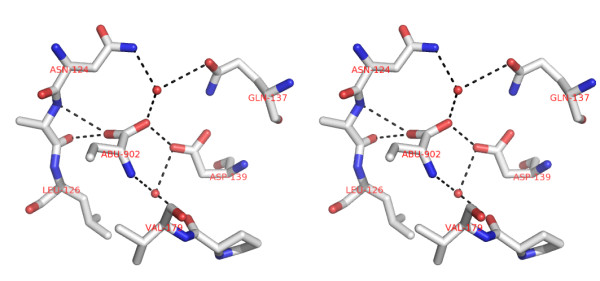
**Stereo view of Abu ligand site**. H-bonds stabilize the Abu (magenta) in the pocket after the anchoring by hydrophobic interactions. The pocket is formed in the monomers interface, which build the canonical dimers of CGL, inside the hydrophobic cavity. Asp 139 from one chain and Ala 125 from the other monomer chain interacts with abu by H-bonds. Two interstitial water molecules (red balls) perform H-bonds with the amino and carboxyl groups of abu. The hydrophobic contacts are established by abu and Leu 126 and Val 179 from the same chain.

Hydrophilic interactions occur between Abu and Asp139 by H-bonds. The OD2 hydroxyl oxygen from Asp139 and Abu oxygen display at 2.4 Å. The same Abu molecule oxygen builds another H-bond with a water molecule at 2.6 Å and the Abu nitrogen also interacts with a water molecule at 2.5 Å (Figure 4).

The residues that compose this hydrophobic pocket are highly conserved in other ConA-like lectins from Phaseoleae tribe, such as, lectins from *Canavalia brasiliensis *seeds (ConBr), *Canavalia maritima *seeds (ConM) and *Dioclea grandiflora *seeds (DGL). These residues were also found to be highly conserved in various lectins from Phaseoleae, Glycineae and Sophoreae tribes from Fabales order. Lectins isolated from seeds of *Bowringia mildbraedii *(BMA), *Dolichos lablab *(Dlab) and *Glicine max *(GML and SBA) presented in their amino acid sequences the same residues that were found in CGL Abu site or, in some cases, an amino acid conservative change (Table 2). All the residues listed form the monomer-monomer interface of the dimmers of these legume lectins. The conservative changes in Abu site, specifically in Ala125, do not seem to be a problem to perform the interaction with molecules like Abu because the hydrogen bond occurs between the Abu and the main chain of this residue. In addition, residues corresponding to Leu 126 and Val 179, which are effective in hydrophobic anchoring of Abu, and the ones corresponding to Asp 139 and Gln 137, responsible by hydrophilic contacts, are highly conserved in spite of the relative phylogenetic distance of these leguminous tribes. It is important to consider that the origin of the genes that codify plant lectin rose from a unique ancestral and the little differences found between them were originated by divergence evolutionary processes [20]. But structures are more conserved than sequences during evolution. Therefore some differences found in the amino acids of Abu site not necessarily mean an absence of functional correlations between the CGL and other lectins [21].

**Table 2:** Amino acids of Abu hydrophobic pocket, showing the conservation in lectins isolated from seeds of leguminous from various tribes.

**Lectin**	**Abu hydrophobic pocket**					
CGL	**Asn 124**	**Ala 125**	**Leu 126**	**Gln 137**	**Asp 139**	**Val 179**
ConA	**Asn 124**	**Ala 125**	**Leu 126**	**Gln 137**	**Asp 139**	**Val 179**
BMA	**Asn 126**	Ser 127	Val 128	**Gln 139**	**Asp 141**	**Val 181**
Dlab	Gln 10	Ser 11	**Leu 12**	**Gln 23**	**Asp 25**	Leu 64
GML	Gln 32	Tre 33	Val 34	**Gln 45**	**Asp 47**	Leu 100
SBA	Glu 02	Tre 03	Val 04	**Gln 15**	**Asp 17**	Ile 57

Bold letters means identical amino acids; and unbolded letters are conservative modifications when compared with CGL.

**Figure 5:**
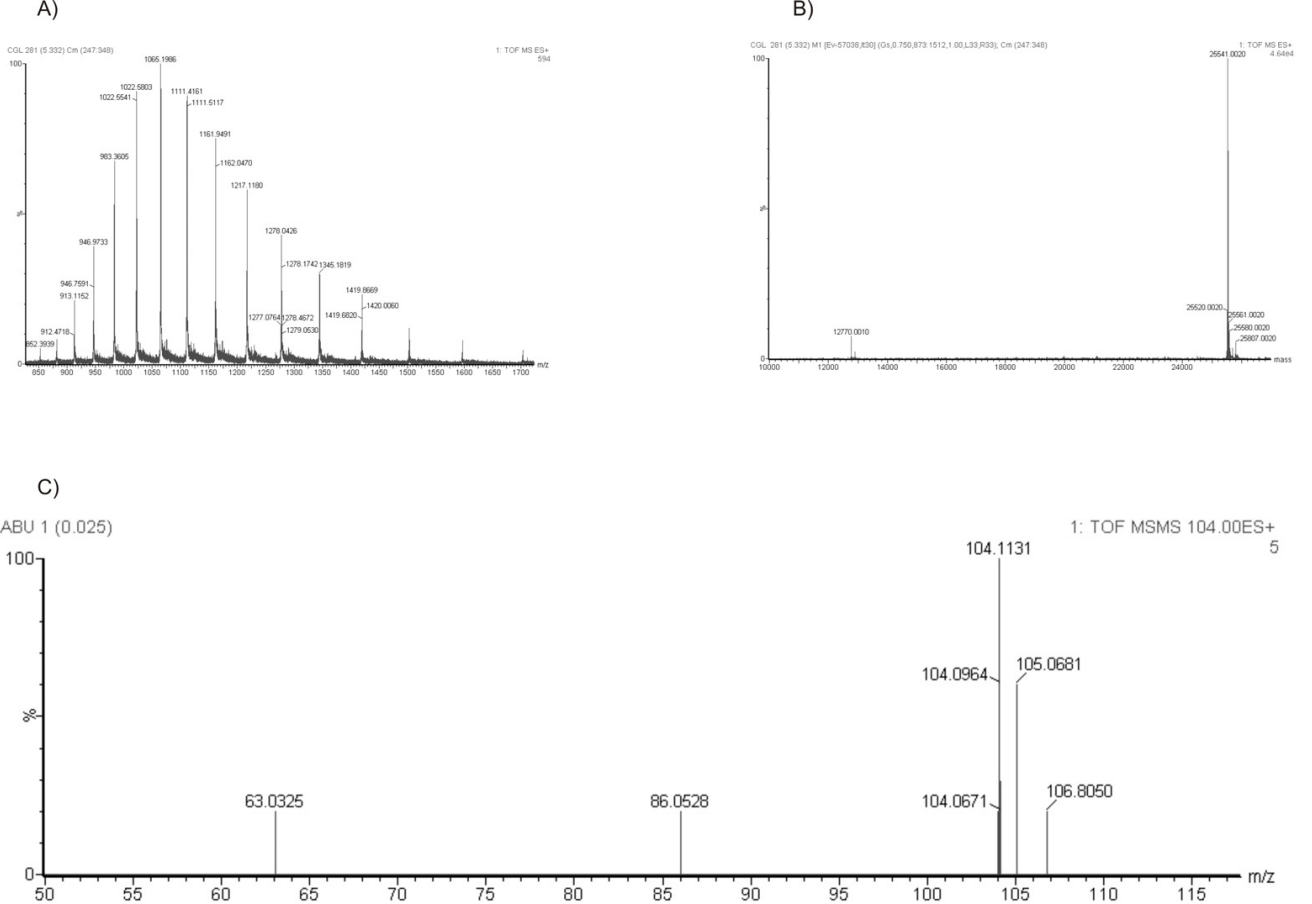
**CGL mass spectrometry analysis**. (A) Mass spectrometry analysis of CGL reveals peaks of ions with charge from +18 (m/z = 1419.8669) to + 28 (m/z = 913.1152). (B) Deconvolution of CGL spectrum, showing the double-charged ion (m/z = 12770.0010) and the mono-charged ion (m/z = 25541.0020) which represents the exact mass of the protein. (C) The Abu signature is represented by the peak with m/z (+H) = 104.1131 ± 0.1 M. The presence of Abu in the CGL structure was confirmed by MS/MS analysis. The spectrum reveals an Abu fragmentation ion displaying m/z = 86.05 ± 0.1, this mass referred to Abu molecular mass with a common loss of a water group (~18 Da).

The mass spectrometry analysis of CGL reveals a very well purified spectrum showing the peaks of ions with charge from +18 (m/z = 1419.8669) to + 28 (m/z = 913.1152) (Figure 5a). The deconvolution of this spectrum reveals two peaks to CGL exact mass; the first peak (m/z = 12770.0010) is the double-charged ion and the second one (m/z = 25541.0020) represents the mono-charged ion and the exact mass of the protein (Figure 5b). The presence of Abu in the CGL structure was confirmed by mass spectrometry. The mass spectra of Abu displayed m/z (+H) = 104.1131 ± 0.1 Min the low mass spectrum in the CGL ESI-Q-ToF MS analysis. The fragmentation of this ion in an MS/MS experiment revealed an ion-fragment with m/z = 86.05 ± 0.1, this mass referred to Abu molecular mass with a common loss of a water group (~18 Da). (Figure 5c).

The finding of Abu in CGL strongly indicates the capability of lectins to bind secondary metabolites. Some legume lectins possess a hydrophobic binding site with high affinity for adenine and certain adenine-derived plant hormones [2]. The binding of adenine has been described mainly for tetrameric legume lectins, including DBL, PHA-E and SBA [3,4]. This new property of lectins supports their role in defense mechanisms in plants.

### Physiological Properties

**Figure 6:**
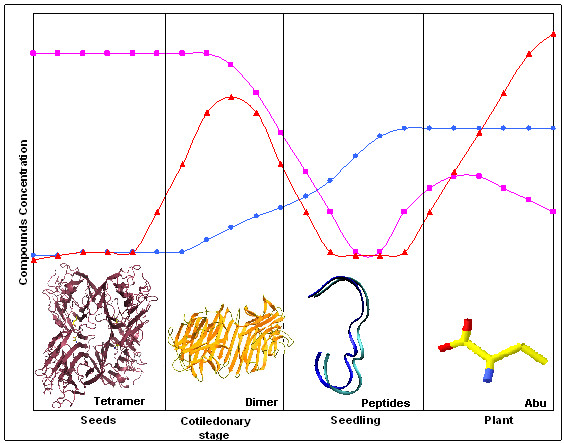
**Schematic graph of the interaction mechanism between the lectin and Abu shows the relation of these molecules and the ISR in leguminous**. The figures inside the frames represent the molecule forms that occur in high concentration during the plant ontogeny. (●) represents the Abu concentration during the seeding as reported by Rozan *et al*.[12]; (■) refers to the lectin concentration determined by Da Silva *et al*.[22]; and (▲) gives the concentration of compounds related to pathogens response and stress events in accord to Boege K & Marquis R J[11].

CGL reveals an interaction site for a non-protein amino acid, which may be involved in defense mechanisms and the induction of response against pathogens in synergy with jasmonate, salicylic acid and abscisic acid pathways. During the final step of the germination of leguminous seeds, Abu concentration increases. In lentil experiments shown by Rozan and collaborators [12], the total content of non-protein amino acids in non-germinating seeds was very low. This content increased after four days of germination and then even more when the stage of seedling was reached [12]. There's a possibility that at least a part of the increase in Abu concentration may be explained by the release of this molecule when the canonical dimmer is disrupted, when the lectins start to be consumed during final steps of germination [22]. Consequently free Abu increases in the seedlings. This hypothesis is schematically depicted in Figure 6.

When Abu concentration increases in seedlings, it promotes the synthesis of ethylene. Once lectins are consumed at the end of germination, it's reasonable to assume that there could be a correlation between lectin-binding substances such as Abu and the development of plant response against stress and pathogens [11,22].

Other non-protein amino acids, such as the Abu isomers β-aminobutyric acid (BABA) and γ-aminobutyric acid (GABA), have been reported to be physiological inducers in many processes related to response-induced resistance and stress [9]. Actually, non-protein amino acids seem to be involved in ethylene-dependent induced systemic resistance [9]. If our hypothesis is true, lectins may have an important role in this mechanism, which would define a lectin-dependent ISR pathway.

It should be highlighted that this discussion above is simply a hypothesis and that we don't really have any certainties regarding the real relevance of this site and the binding of Abu to the lectin. But the fact that the amino acids present in this site are conserved in legume lectins as mentioned before is undoubtedly an evidence that an important biological activity is mediated by this site.

The common carbohydrate-binding site of lectins has been reported as the main target in the study of lectins, but evidence of new biological sites leads to questions about the physiological function of these proteins, first considered storage proteins and later found to play a role in plant defense mechanisms.

The increase in Abu concentration in the seedling corresponds to the increasing induction of hypersensitive response in adult plants considering the high concentrations of HR inducers at this time of plant ontogeny as described by Boege and Marquis [11]. In agreement, lectin concentration in seeds declines drastically by the end of germination and, therefore, is lower in the seedling (Figure 6).

## Conclusion

Our study supports other investigations proposing a function for lectins beyond simple carbohydrate recognition. Plant lectins are proteins which may also interact with secondary metabolites, displaying cell recognition, and defense functions in plant physiology. Here, we described a new interaction site not related to the sugar-binding site in a ConA-like lectin that may be related to plant defense.

## Methods

### Crystallization and Data Collection

The purification of Canavalia gladiata lectin (CGL) was performed as discribed by Cecatto *et al*. [23] and Moreno *et al*. [24]. Crystals of the *Canavalia gladiata *lectin were grown by the hanging drop vapor diffusion method, drops contained 3 μL of protein solution and 3 μL of 0.1 M Tris-HCl, pH 8.5, with 2.0 M ammonium sulfate. Cryoprotected native CGL crystals diffracted to a resolution of 2.3 Å using a synchrotron radiation source and indexed, integrated and scaled at the same resolution. Low resolution data sets have been collected in capillar without cryoprotection at 2.9, 3.0 and 3.1 Å resolution in room temperature to confirm the presence of Abu. Native crystals were also soaked with a solution containing 1 mM α-methyl-mannoside. The complexed crystals diffracted to 2.3 Å. The data were indexed, integrated and scaled at 2.3 Å using MOSFLM [25] and SCALA [26]. Crystals belong to the orthorhombic space group *C222*_1_. The calculated Matthews coefficient indicated a tetramer in the asymmetric unit, and this protein assumes the same oligomerization in biological conditions.

### Molecular Replacement and Refinement

The crystal structures CGL and CGL-αMM were determined by molecular replacement using the MolRep program [26]. *Canavalia ensiformis *lectin (ConA) structure without the complexed sugar and water molecules (PDB code 5CNA) was used as the search model [16]. The Abu coordinates were obtained by PRODRG program [27]. The initial structures were refined using REFMAC5 [28] and water molecules were added to the models using XtalView [29]. The crystal structures were deposited in the PDB [30] with accession code 1 WUV and 2D7F. The crystallographic statistics data are shown in Table 1. An omit map contoured at 3 σ to the aminobutyric acid was generated using CCP4 Omit program [28].

### Mass Spectrometry Analysis

The presence of Abu in the molecule of native CGL was analyzed in an ESI-Q-ToF/MS/MS experiment. An amount of 1 mg of CGL was dissolved in 1 mL of a solution containing 50% acetonitrile and 5% TFA. The protein solution was diluted 100 × and injected in a nano-electrospray ionization source and analyzed in a Micromass™ quadrupole time-of-flight instrument with a resolution of 8,000 and accuracy of 10 ppm. The spectral data were processed using a Biolynx 4.0 program.

## Abbreviations used

Abu, α-aminobutyric acid; CGL, *Canavalia gladiata *lectin; HR, Hypersensitive response; CGL-αMM, *Canavalia gladiata *lectin in complex with α-methyl-mannoside; ConA, *Canavalia ensiformis *lectin; ESI, Electrospray ionization source; Q-ToF, Quadrupole-time of flight analyzer; SAR, systemic acquired resistance; ISR, induced systemic resistance.

## Authors' contributions

PD solved the x-ray crystallography structure of CGL in complex with a-methyl-mannoside, carried out Synchrotron x-ray data collection, and drafted the manuscript. BAMR carried out the mass spectrometry analysis, established the physiologic correlations of Abu, helped in the x-ray crystallography studies and Synchrotron x-ray data collection, and drafted the manuscript. EPS helped in the x-ray crystallography studies and Synchrotron x-ray data collection. TMO carried out the purification experiments, and drafted the manuscript. GAB carried out the purification experiments, and drafted the manuscript. FBMBM crystallized the CGL and helped in Synchrotron x-ray data collection. BTF solved the x-ray crystallography structure of native CGL. TSG carried the prospection of the possible molecules found in seeds and which can interact with the lectin. AHS helped in the x-ray crystallography studies and Synchrotron x-ray data collection, and drafted the manuscript. WFA conceived the study, and participated in the design and coordination. BSC conceived the study, and participated in the design and coordination.
